# Supplementary material for: Federated epidemic surveillance
Source: PLoS Comput Biol. 2025 Apr 8;21(4):e1012907. doi: 10.1371/journal.pcbi.1012907 (PMC11978105; doi:10.1371/journal.pcbi.1012907)
Supplement: S1 Appendix — (PDF) [file pcbi.1012907.s001.pdf]

Supplementary Material for "Federated Epidemic Surveillance"

Ruiqi Lyu[1*], Roni Rosenfeld[2], Bryan Wilder[2]

1 Computational Biology Department, Carnegie Mellon University, Pittsburgh, Pennsylvania, United States of America
2 Machine Learning Department, Carnegie Mellon University, Pittsburgh, Pennsylvania, United States of America

* ruiqil@cs.cmu.edu

Parameters and notations

Here is a brief introduction to the notations used in this article. These notations are summarized in Table 1.

Table 1. Parameters and notations.

**Distribution**	
k	Poisson distributed count
λ	Poisson rate parameter
c	Binomial distributed count
n	Binomial success probability
ρ	Binomial number of trials
**Hypothesis Test**	
θ	Null growth rate
θ'	Real growth rate
α	Type I error rate
α'	Calibrated confidence level for different methods
l	Relative length of the baseline period
N	Number of sites
s	Shares of different sites
w	Weights for combining p-values
**Subscript**	
i	Index of the sites ($i \in \{1, \ldots, N\}$)
j	Index of the baseline period ($j \in \{1, \ldots, l\}$)
B, T	Indicator of baseline period and testing period

Power computation

The power of a hypothesis test is the probability that the test correctly rejects the null hypothesis H_0 when a specific alternative hypothesis H_1 is true. In our case when conducting the binomial test under the null hypothesis $H_0 : \frac{\lambda_T}{\lambda_T + l\lambda_B} \leq \frac{1+\theta}{1+\theta+l}$, it is necessary to determine the critical value k_{cr} for k_T at a specific confidence level α. This critical value represents the minimum number of successes in the sample that would reject the null hypothesis in favor of the alternative hypothesis. Notably, the actual

confidence level of the discrete distribution is strictly less than α because the critical value must be an integer. Equation 1 is used to determine the critical value.

$$\begin{aligned}\alpha \quad &\geq \Pr(r \geq k_{cr} | n = \textstyle\sum_{j=1}^{l} k_{Bj} + k_T, \frac{1+\theta}{1+\theta+l}) \\ &= \textstyle\sum_{r=k_{cr}}^{n} \binom{n}{r} \frac{(1+\theta)^r \cdot l^{n-r}}{(1+\theta+l)^n}\end{aligned} \tag{1}$$

Under the alternative hypothesis, where the underlying growth rate is $\theta' > \theta$, the power is computed as shown in Equation 2.

$$\begin{aligned}\text{power} \quad &= \Pr(r \geq k_{cr} | n = \textstyle\sum_{j=1}^{l} k_{Bj} + k_T, \frac{1+\theta'}{1+\theta'+l}) \\ &= \textstyle\sum_{r=k_{cr}}^{n} \binom{n}{r} \frac{(1+\theta')^r \cdot l^{n-r}}{(1+\theta'+l)^n}\end{aligned} \tag{2}$$

Even though the Binomial distribution yields discrete values, we can simplify the computations within an acceptable range by applying a Gaussian approximation with a continuity correction. Assuming the null hypothesis, the count distribution during the testing period can be approximated by a Gaussian distribution with mean $\frac{n(1+\theta)}{1+\theta+l}$ and standard deviation $\frac{\sqrt{nl(1+\theta)}}{1+\theta+l}$. The critical value can then be calculated using the expression $\tilde{k}_{cr} = \frac{n(1+\theta)}{1+\theta+l} + \frac{Z_\alpha\sqrt{nl(1+\theta)}}{1+\theta+l}$, where Z_α is the α level's z-score. We can then compute the power under the alternative hypothesis using Equation 3.

$$\begin{aligned}\text{power} \quad &= \Phi\Big(-\frac{k_{cr} + \frac{1}{2} - \frac{n(1+\theta')}{1+\theta'+l}}{\frac{\sqrt{nl(1+\theta')}}{1+\theta'+l}}\Big) \\ &= \Phi\Big(\frac{\sqrt{nl}(\theta'-\theta)}{(1+\theta+l)\sqrt{(1+\theta')}} - \frac{Z_\alpha(1+\theta'+l)\sqrt{1+\theta}}{(1+\theta+l)\sqrt{(1+\theta')}} - \frac{1+\theta'+l}{2\sqrt{nl(1+\theta')}}\Big)\end{aligned} \tag{3}$$

The expression inside the $\Phi(\cdot)$ function consists of three terms, each representing a specific aspect of the analysis. The first term quantifies the impact of the total count magnitude, while the second term relates to the confidence level. The third term corresponds to the continuity correction term, which can be ignored when the sample size n is sufficiently large.

Power curves and calibrations

In the main text, we compute and compare the statistical power of centralized and decentralized hypothesis tests using different meta-analysis methods. Those methods exhibit different properties in combining p-values, which is apparent when examining the frequency of rejecting the null hypothesis. As the real growth rate, θ', increases, so does the number of rejections for all tests. The rejection rate under the null hypothesis is termed the false positive rate (FPR), while under the alternative hypothesis, it is known as the power.

To maintain the FPR of each meta-analysis method below a predetermined threshold of $\alpha = 0.05$ under the null hypothesis, we adjust the confidence levels, α', of these methods. This adjustment is achieved through numerical experiments and ensures that α' corresponds to the α quantile of the combined p-values' distribution. Figure 1 shows the calibrated confidence levels α' for different methods under different magnitudes of total counts. This calibration process controls the likelihood of committing Type I errors (erroneously rejecting a true null hypothesis), keeping it at the desired level of α. Statistically, the threshold α' signifies the point where the cumulative distribution function (CDF) of the combined p-values reaches α. The analysis reveals that Stouffer's and Pearson's methods typically yield conservative p-values, whereas other methods often result in significantly smaller combined p-values. As the number of sites increases and the reported total counts decrease, this disparity becomes increasingly significant.

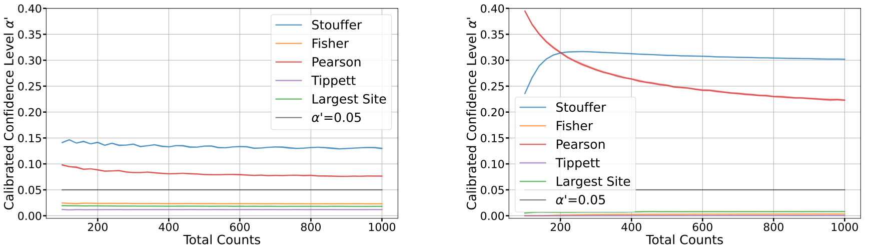

(a) Two sites provide the p-values. **(b)** Eight sites provide the p-values.

Fig 1. Calibrated the confidence level α'.

The main text presents the power curves for various meta-analysis methods. Additionally, Figure 2 illustrates the modified Fisher's method and Stouffer's method enhanced with auxiliary information. As the distribution of shares between two facilities becomes more unbalanced, the performance of traditional combination methods is constrained. However, modifications such as weighting significantly improve robustness to such imbalances. The weighted and continuity-corrected Stouffer's methods are particularly effective in approximating the binomial power.

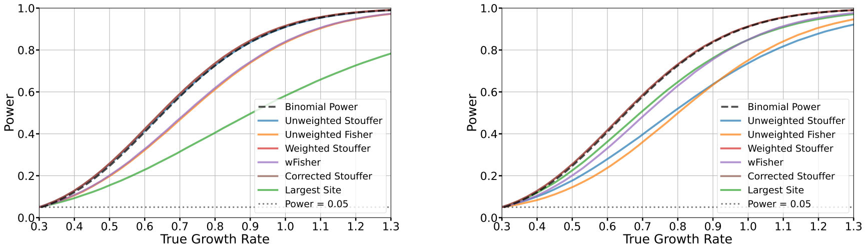

(a) Two sites provide the p-values and the larger has 60% share. **(b)** Two sites provide the p-values and the larger has 90% share.

Fig 2. Power analysis of the modified methods.

Normalized entropy in Semi-synthetic analysis

In our semi-synthetic analysis, we fixed the number of sites at $N = 5$ and constructed share distributions that range from highly imbalanced (one site dominating) to perfectly balanced (all sites equal). We use the normalized entropy metric $S = \frac{-\sum_{i=1}^{N} s_i \log(s_i)}{\log(N)}$ to measure how evenly shares are distributed. A value of $S = 1$ indicates perfectly equal shares, while lower values reflect greater imbalance. Table 2 shows the share distributions and their corresponding entropy values with fixed the number of sites at $N = 5$. Note that while entropy helps summarize the degree of balance, it is the imbalance of the shares themselves, not the entropy per se, that drives differences in power.

In addition, Figure 3 illustrates how entropy and the magnitude of counts together influence performance. The first panel shows results for Stouffer's method, the second for Fisher's method, and the third displays their difference. Although the difference is small due to the limited range of variation available in real data, a clear trend emerges: both methods perform worse when counts are low and shares are imbalanced, but Fisher's method remains more robust under these challenging conditions. In contrast,

Share	Entropy
$0.05, 0.05, 0.05, 0.05, 0.80$	0.4832
$0.05, 0.05, 0.05, 0.10, 0.75$	0.5563
$0.05, 0.05, 0.10, 0.10, 0.70$	0.6274
$0.05, 0.10, 0.10, 0.10, 0.65$	0.6963
$0.10, 0.10, 0.10, 0.10, 0.60$	0.7627
$0.10, 0.10, 0.10, 0.15, 0.55$	0.8103
$0.10, 0.10, 0.10, 0.20, 0.50$	0.8445
$0.10, 0.10, 0.10, 0.30, 0.40$	0.8814
$0.10, 0.10, 0.20, 0.20, 0.40$	0.9139
$0.10, 0.10, 0.20, 0.30, 0.30$	0.9350
$0.10, 0.20, 0.20, 0.20, 0.30$	0.9675
$0.20, 0.20, 0.20, 0.20, 0.20$	1.0000

Table 2. Examples of share distributions for $N = 5$ and their corresponding normalized entropy values.

when the shares are more evenly balanced, Stouffer's method tends to achieve better performance.

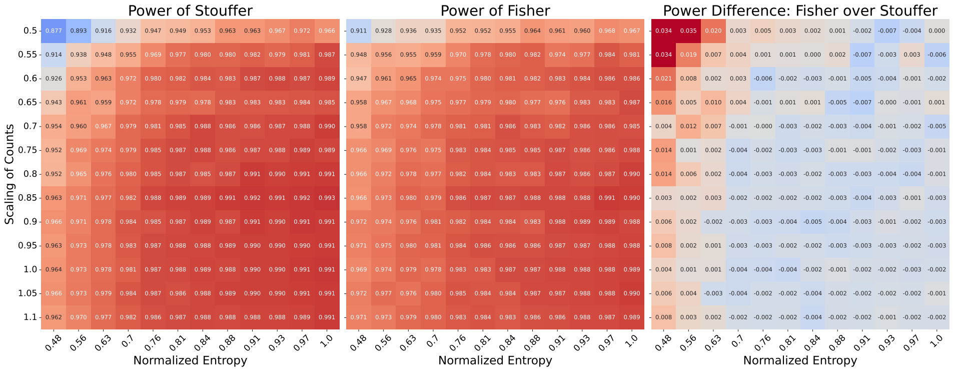

Fig 3. Semi-synthetic analysis using Stouffer's method and Fisher's method.

Proof for Equation 10

On the right hand side, we simply use the Chernoff bound, where we won't provide a proof. On the left hand side, we first prove Lemma 1 inequality based on [1].

Lemma 1. *If* $0 < c < n$, *then*

$$\frac{\exp\left(nH(\frac{c}{n})\right)}{\sqrt{8c(n-c)/n}} \leq \binom{n}{c} \leq \frac{\exp\left(nH(\frac{c}{n})\right)}{\sqrt{2\pi c(n-c)/n}}$$

where the $H(\frac{c}{n})$ *is the entropy of variable* $\frac{c}{n}$, *defined by* $H(\frac{c}{n}) = \frac{c}{n}\log(\frac{c}{n}) + \frac{n-c}{n}\log(\frac{n-c}{n})$

Proof. According to Stirling's formula

$$n! = \sqrt{2\pi n} n^n e^{-n} \exp\left(\frac{1}{12n} - \frac{1}{360n^3} + \cdots\right)$$

To establish the left-hand inequality, since $n! \geq \sqrt{2\pi n} n^n e^{-n}$, $c! \leq \sqrt{2\pi c} c^c e^{-c} \exp\left(\frac{1}{12c}\right)$, we have

$$
\begin{aligned}
\binom{n}{c} &= \frac{n!}{c!(n-c)!} \\
&\geq \frac{\sqrt{2\pi n} n^n e^{-n} \exp(-\frac{1}{12c} - \frac{1}{12(n-c)})}{\sqrt{2\pi c} c^c e^{-c} \sqrt{2\pi(n-c)}(n-c)^{(n-c)} e^{-(n-c)}} \\
&= \frac{\exp(-\frac{1}{12c} - \frac{1}{12(n-c)})}{\sqrt{\frac{2\pi c(n-c)}{n}}(\frac{c}{n})^c(\frac{n-c}{n})^{(n-c)}}
\end{aligned}
$$

By the symmetry in c and $n-c$ of the inequality, without loss of generality, we assume $c \leq n-c$ (otherwise we will exchange the notation of c and $n-c$). We also assume that $c \geq 1$ and $(n-c) \geq 3$. The only remaining cases to consider are $c = 1, n-c = 1; c = 1, n-c = 2; c = 2, nq = 2$. For these cases, the inequality may be verified by direct substitution. So we have

$$
\begin{aligned}
\frac{1}{12c} + \frac{1}{12(n-c)} &\leq \frac{1}{12} + \frac{1}{36} = \frac{1}{9} \\
\exp\left(-\frac{1}{12c} + \frac{1}{12(n-c)}\right) &\geq e^{-\frac{1}{9}} > \frac{1}{2}\sqrt{\pi}
\end{aligned}
$$

Hence

$$
\binom{n}{c} \geq \frac{(\frac{c}{n})^{-c}(\frac{n-c}{n})^{-(n-c)}}{\sqrt{8c(n-c)/n}} = \frac{\exp\{-n[\frac{c}{n}\log(\frac{c}{n}) + \frac{n-c}{n}\log(\frac{n-c}{n})]\}}{\sqrt{8c(n-c)/n}} = \frac{\exp\left(nH(\frac{c}{n})\right)}{\sqrt{8c(n-c)/n}}
$$

which proves the left-hand inequality.

To establish the right-hand side, we use $n! \leq \sqrt{2\pi n} n^n e^{-n} \exp\left(\frac{1}{12n}\right)$, $c! \geq \sqrt{2\pi c} c^c e^{-c} \exp\left(\frac{1}{12c} - \frac{1}{360c^3}\right)$

$$
\binom{n}{c} \leq \frac{\sqrt{2\pi n} n^n e^{-n} e^{\frac{1}{12n}}}{\sqrt{2\pi c} c^c e^{-c} \sqrt{2\pi(n-c)}(n-c)^{(n-c)} e^{-(n-c)} \exp\left(\frac{1}{12c} - \frac{1}{360c^3} + \frac{1}{12(n-c)} - \frac{1}{360(n-c)^3}\right)}
$$

Without loss of generality, we still assume $c \leq n-c$; then $\frac{1}{360c^3} \leq \frac{1}{360c}$ since $c \geq 1$; similarly $\frac{1}{360(n-c)^3} \leq \frac{1}{360(n-c)}$.

Thus we obtain

$$
\begin{aligned}
&\frac{1}{12n} - \frac{1}{12c} - \frac{1}{12(n-c)} + \frac{1}{360c^3} + \frac{1}{360(n-c)^3} \\
&\leq -\frac{1}{12c} + \frac{1}{360c} + \frac{1}{360(n-c)} \\
&\leq -\frac{1}{12c} + \frac{1}{180c} \leq 0
\end{aligned}
$$

Hence

$$
\binom{n}{c} \leq \frac{(\frac{c}{n})^{-c}(\frac{n-c}{n})^{-(n-c)}}{\sqrt{2\pi c(n-c)/n}} = \frac{\exp\{-n[\frac{c}{n}\log(\frac{c}{n}) + \frac{n-c}{n}\log(\frac{n-c}{n})]\}}{\sqrt{2\pi c(n-c)/n}} = \frac{\exp\left(nH(\frac{c}{n})\right)}{\sqrt{2\pi c(n-c)/n}}
$$

which completes the proof.

□

With Lemma 1, we can subsequently prove the inequality in Lemma 2.

Lemma 2.

$$p\text{-value} \geq \frac{1}{\sqrt{\frac{8}{n}c(n-c)}} \exp(-nD(\frac{c}{n}\|\rho))$$

Proof.

$$\begin{aligned}
p\text{-value} = \mathrm{CDF}_{\mathrm{Bin}}(c; n, \rho) &= \sum_{r=0}^{c} \binom{n}{r} (1-\rho)^{n-r}\rho^{r} \\
&\geq \binom{n}{c}(1-\rho)^{n-c}\rho^{c} \\
&= \binom{n}{c} \exp\{-n(\frac{n-c}{n}\log\frac{1}{1-\rho} + \frac{c}{n}\log\frac{1}{\rho})\} \\
&= \binom{n}{c} \exp\{-n[D(\frac{c}{n}\|\rho) + H(\frac{c}{n})]\} \\
&\geq \frac{\exp\left(nH(\frac{c}{n})\right)}{\sqrt{8c(n-c)/n}} \exp\{-n[D(\frac{c}{n}\|\rho) + H(\frac{c}{n})]\} \\
&= \frac{1}{\sqrt{\frac{8}{n}c(n-c)}} \exp(-nD(\frac{c}{n}\|\rho))
\end{aligned}$$

□

Summary of ground truth windows

Each ground truth surge is surrounded by a specified time window, within which a reconstructed alert is considered a true positive. The "true" surges are determined as described in the main text, and each ground truth window spans from one week before to two weeks after a detected surge. Figure 4 displays the coverage proportions that show how much of the overall timeline is encompassed by these ground truth intervals around true surges.

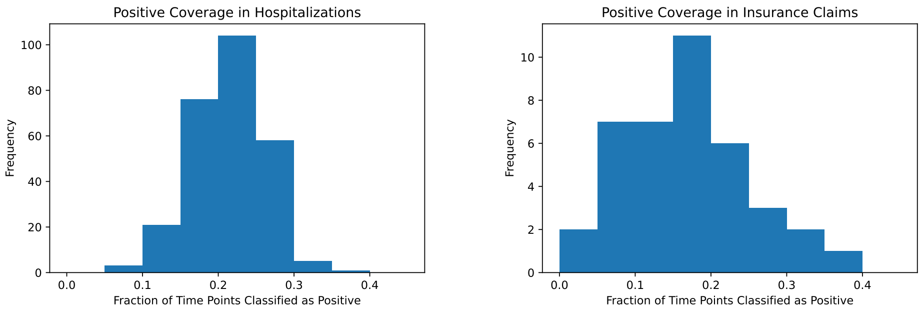

Fig 4. Coverage of ground truth across the whole time period for different datasets.

Detection delay

We present the distribution of detection delays as Figure 5 and Figure 6 at a p-value threshold equals to 0.05, revealing how many detections occur early or late relative to the true surge dates.

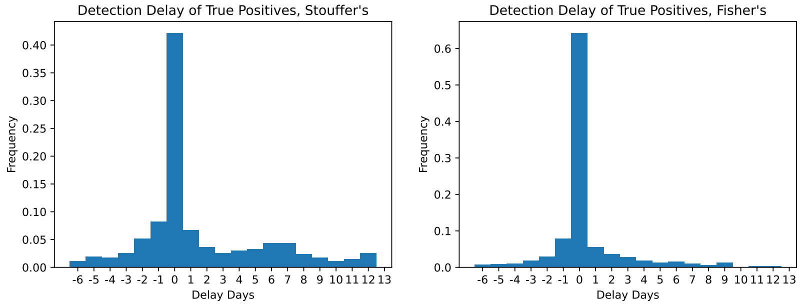

Fig 5. Detection delay days for true positives in insurance claims.

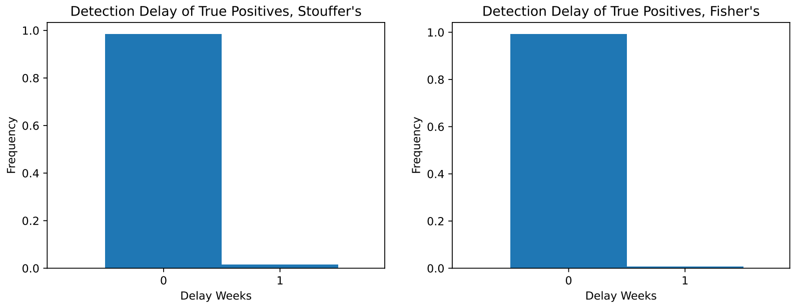

Fig 6. Detection delay weeks for true positives in hospitalizations.

References

1. Ash RB. Information theory. Courier Corporation; 2012.
