# Supplementary material for: Federated epidemic surveillance
Source: PLoS Comput Biol. 2025 Apr 8;21(4):e1012907. doi: 10.1371/journal.pcbi.1012907 (PMC11978105; doi:10.1371/journal.pcbi.1012907)
Supplement: Attachment — Submitted filename: LetterJan2025.pdf [file pcbi.1012907.s004.pdf]

We thank the reviewers for their insightful comments. We have updated the manuscript accordingly. Here are our responses:

Reviewer #1:

First, this work is only relevant if implemented. It can only be implemented if the research community gets buy-in from data custodians or if mandatory reporting guidelines are shifted to include p-values for certain targets. Both of these are big tasks. Could you write to that in the discussion? Maybe you've thought of additional ways to get this data, but as you well know, it's hard to get raw data from facilities for all of the reasons you mentioned in the paper. I can't imagine that many administrators are going to be amenable to running some quick stats to send to researchers. Do you have any ideas about how to get a system like this up and running?

Thank you for your question, we agree it is an important question and is often discussed when we present the work. According to what we discussed with health facilities and departments, they are open to trying the framework and believe transferring summary statistics is easier than sharing the counts. However, they also argue that it's more possible to implement the framework on an easy-to-use, standardized software tool for some agreed-upon hypothesis tests not limited to Poisson counts. To the Discussion section, we have added the sentence (in Line 650) "Implementation will likely rely on mandatory reporting guidelines, alongside easily-usable software for agreed-upon hypothesis tests, not limited only to Poisson counts.".

Second, could you include a two-panel heatmap of entropy vs. magnitude of counts, with one panel for Stouffer and one for Fisher (even if it needs to be in a supplement)? I think the unweighted methods are probably sufficient.

Thanks for the suggestion. The plots are attached and mentioned (in Line 529) in the main text, and we added the "Normalized entropy in Semi-synthetic analysis" of Supplementary Material. Of note, It's the imbalance of the share that substantially causes the difference of powers, rather than the entropy. We used the entropy only to evaluate the imbalance of the share after fixing the number of sites.

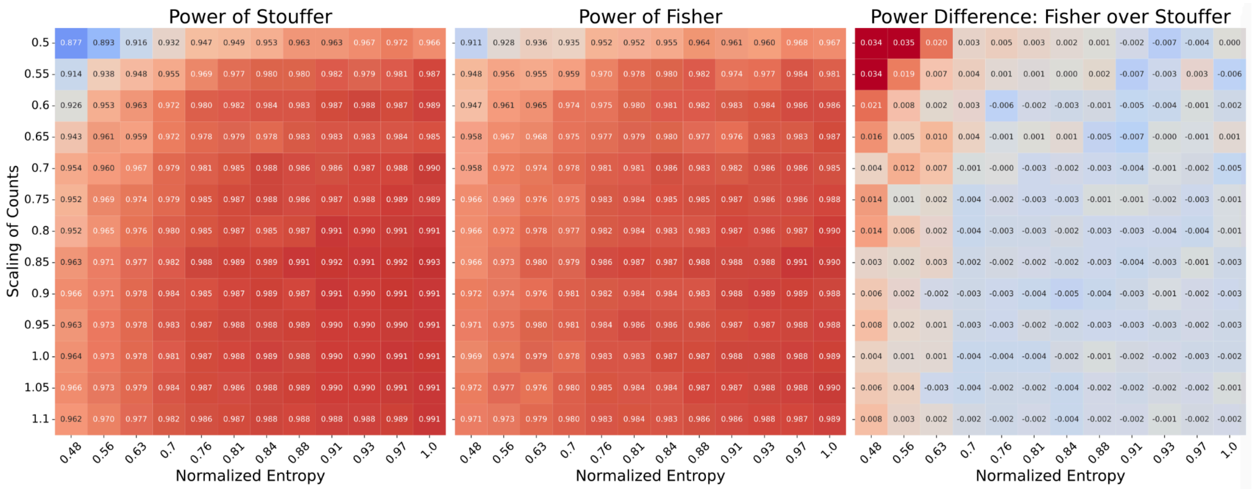

There are more details in the comments in the PDF.

Thanks for your attention to detail. We resolved the comments in the manuscript accordingly.

I don't know the answer to this, but is backfill an important issue to consider here? Does backfill happen within facilities as well as in mandatory reporting of certain disease targets in larger datasets like the NHSN dataset? If so, this should be mentioned as a limitation in the discussion.

It's a good question. Many applications of our framework could involve data subject to backfill, in which case the hypothesis test of interest may incorporate some correction for backfill (e.g., waiting some amount of time for counts to stabilize, or performing hypothesis tests on a projection of what the final number of cases will be). While optimally handling backfill is outside the scope of this paper, the need to do so could reduce the detection speed or statistical power of surge detection. We added "Addressing the temporal patterns of reporting delays such as backfill may further increase power." to the Discussion section (Line 643).

Reviewer #2:

This is a well written and comprehensive look at an important and novel area of epidemic science. My overall impression is that this manuscript covers a lot of ground, and could benefit from moving some sections to supplemental materials (eg deep dives on methods: pages 11-14) . Additionally, an architectural diagram of the abstract process (or one that is more detailed describing the different study designs) would help a bit, as there are a lot concepts to entertain and only detailed plots of data to guide the reader. If not already planned a companion article describing the general proposed solution and its applications with less focus on methodology would be very useful to the community writ large.

We thank the reviewers for going over the methodology in detail.

Regarding moving some sections of the methods, we have elected to keep the sections on Stouffer's and Fisher's as they are highly relevant to the overall methodology of the paper and demonstrate the general usage across different tasks, as we provided limited scenarios (e.g., distributional assumptions, hypothesis tests, etc.) for the proof of concept. This information is relevant to understanding the work as is, as well as possible extensions.

We added the flowchart below to the problem formulation section of the methods.

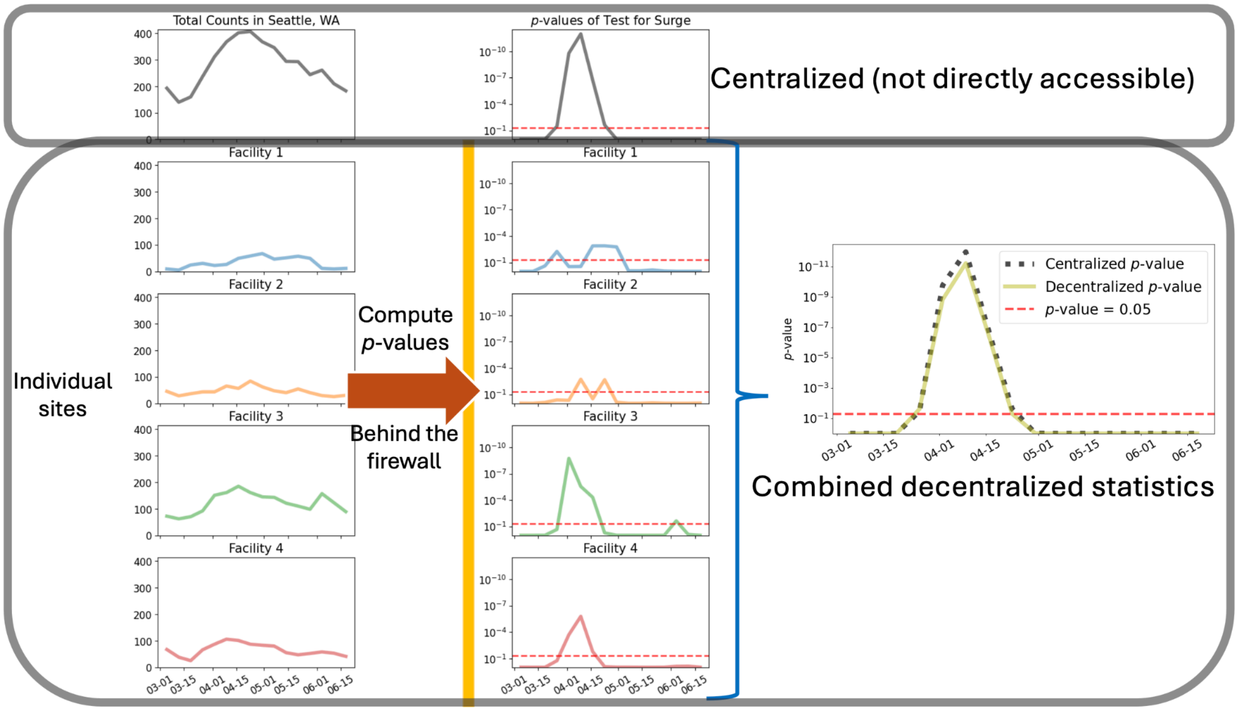

I understand that this is intended to be abstract and applicable to a wide range of possible applications. However, a lot of the paper is spent on evaluation (as it should be). I am quite familiar with the datasets described, however, it is not exactly clear the overall size of the datasets (eg number of observations, total number of facilities etc) used in these analyses. Figure 2 provides one perspective of the data, but still lacks some of these simple qualitative descriptions. For instance how many aggregate level “true” surges are there?

Thanks for the suggestion. The number of observations is indicated by the length of the period, as we have the weekly/daily reports. The weekly hospitalization covers the period from 2020-07-10 to 2023-03-03, and the insurance claim covers the period from 2020-08-02 to 2022-07-30. We added below to the main text Line 62: “We combine the summary statistics from 769 facilities from 286 metro-areas in the hospitalization dataset, and the summary

statistics from 427 counties from 39 states in the insurance claim dataset. For decentralized analysis, we filter hospitalization summary statistics with more than 10% missing or 0 values, and insurance claim summary statistics with more than 50% missing or 0 values. We note these facilities still play a role in constructing the centralized ground truth. We also do not analyze single-site regions, as the decentralized and centralized statistics are the same."

The aggregate level "true" surges vary depending on the dataset in our experiment, and we filtered those that have no surges out of our analysis. For the hospitalization dataset, the mean and the standard deviation of the number of surges in each aggregate level series are 15.82 and 3.87; for the insurance claim dataset, the mean and the standard deviation are 19.51 and 16.20. We added this description to Line 353. More detailed descriptions of ground truth are in the Supplementary note (Summary of ground truth windows).

Embedded in the detailed description of the methodology is that the main task is to detect a surge at aggregate levels based on the p-values of the surge detection statistic at the individual sites. I assumed, incorrectly, based on the datasets, that this would be state level from facilities and state level from counties (HHS and Change healthcare claims respectively). It's not till page 15 in the description of the data generation that the word "state" is used, and then on page 18 the scale and level of the task comes into focus when the exact comparison is spelled out: "we add up the counts of facility-level hospitalization to generate county-level p-value alerts, and county-level insurance claim counts to create state-level p-value alerts"

Yes, you are right, thanks. We added the sentence "We accumulate facility-level hospitalization counts to consider metro-area-level detection, and county-level insurance claim counts for state-level detection." to the Datasets section (Line 59).

Perhaps an overview of the proposed evaluations earlier on in the materials and methods would benefit other readers (this would be more clear too with quick description of the data suggested above as well). I think this would also help orient readers to the various stages of the evaluation. As is, the authors provide a nice narrative and gently guide the reader through the successive levels of evaluation, however, given the depth and length a summary up front would be helpful.

Thanks for the suggestion. We added the sentence "We evaluate the success of our proposed framework by its ability to reconstruct the signal present in the fully centralized setting, where all count information is available, or by recapitulating the signals indicated by semi-synthetic growth rates." to the start of the Problem Formulation section of the methods (Line 76), alongside the description of the data above.

In the evaluation of the surge detection task section there is an important assumption about what is "true" vs. "false" positive in lines 324-328. A one month window seems generous, though in practice 2 weeks after is still useful especially if true ground truth data is not knowable, so I think this is "reasonable." Interpretation of the findings would

benefit from understanding the proportion of all time points that are covered by these windows around true surges vs. not covered. A distribution of how many of these "true positives" come 1 week early vs. 2 weeks later is interesting as well. There are a lot of sensitivities explored in this paper however the impact of this definition seems important and seems to be left unexplored.

Thank you for your advice. We added more detailed descriptions of ground truth windows to in Summary of ground truth windows and detection delays to Detection delay sections of Supplementary Material.

The coverages of the true positive windows around the surges are plotted below. On average, around 20% of the total region is considered a ground truth, and a maximum of 40%, for both hospitalizations and insurance claims. A section discussing this figure has been added to the appendix.

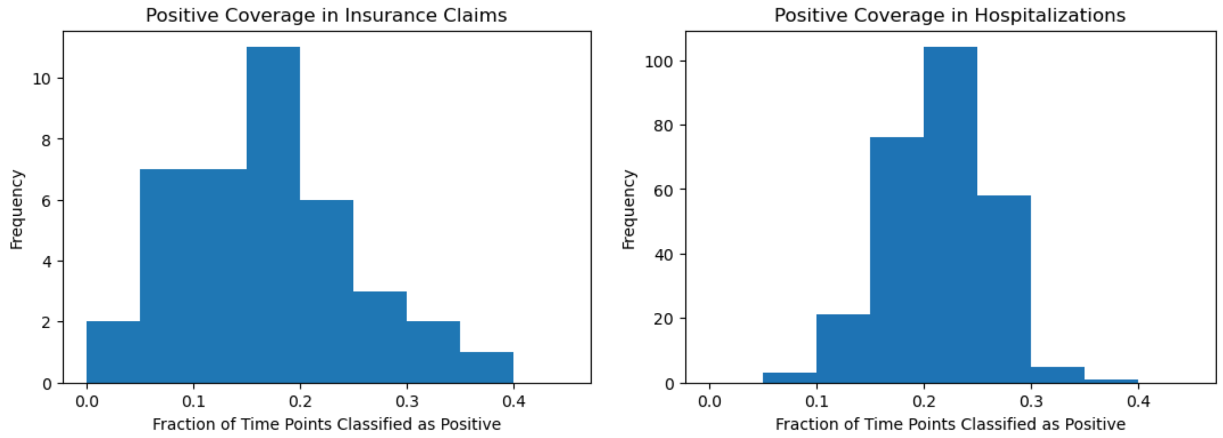

Detection delay of insurance claims with a p-value threshold of 0.05 are plotted here, and has also been added to the Supplementary Material, and the following added to Line 459 "In insurance claim data, we observe 71.1% and 88.9% of true positives detected within 3 days of the true surge for Stouffer's and Fisher's methods, respectively. As hospitalizations are reported weekly, we observe 98.4% and 99.2% of true positives detected at the week of the true surge for Stouffer's and Fisher's methods, respectively. Figures of the distribution of detection days or weeks are shown in Detection Delay section of Supplementary Material." Based on these

results, we expect very little sensitivity to changes in the width of the “true positive” window.

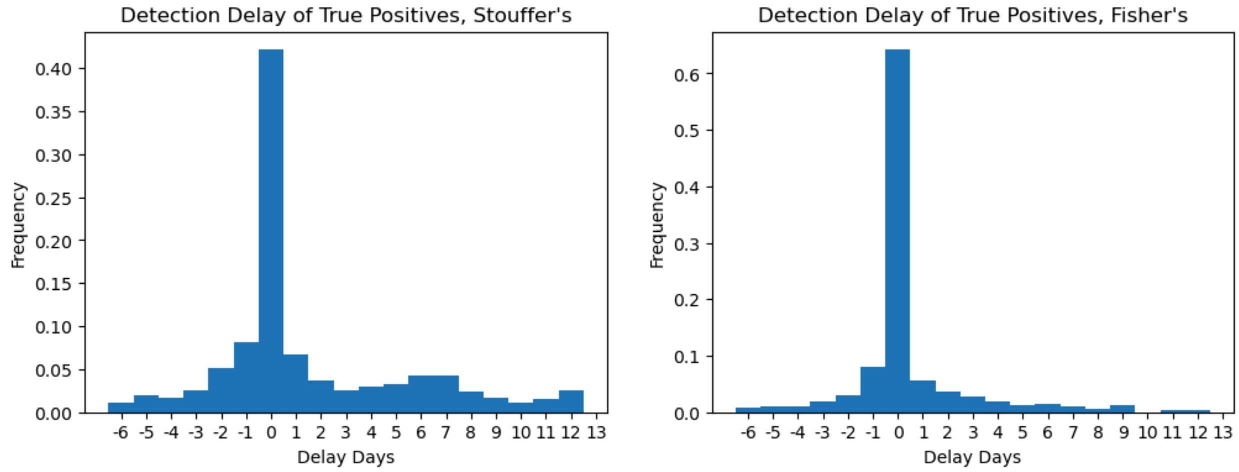

As the hospitalization data is reported weekly and has a smaller window size, it has > 90% zero delay.

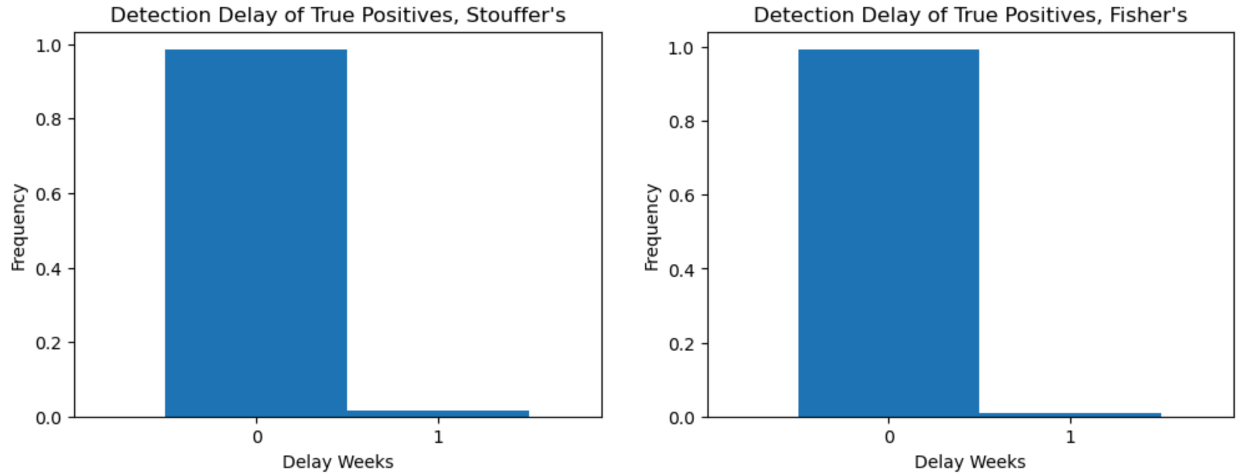

Review of the code helpfully provided on github with embedded data was useful, however with the limited documentation I was not able to get the scripts to run. I imagine there is a specific order that the analyses build off of and some dependencies that I may not have (though none I initially saw). Some of the errors suggested that there may be some directory structures hard-coded that would make this not possible to run outside of the author’s personal environment (eg plot_amp_rewrite.py) Some documentation about what each script does and/or protocol is needed to make these results reproducible.

The code has been made runnable and the readme file was updated, thank you for noting this.

Lines 423-5: This is stated many times and is important. Details on how many sites and importantly “how they were chosen” should be included somewhere. I am looking at PA_Scranton.csv in the data repo and don’t see facility 390119, which is the example

facility on the delphi page for this dataset. Why was this facility not included when its address is in Scranton?

It was filtered from the input to our algorithms for the decentralized setting because 65% of the reports are zero. Importantly, though the facility 390119 is not used by the decentralized methods, , it contributes to the total counts (and therefore centralized setting) in PA_Scranton.csv, like the last two lines of the file, facility 390001 + facility 390237 + filtered_facilities = total_cnts . Filtered facilities are not included in the file.

The case of a single facility is described before the motivation of its inclusion (lines 448-9) which is a little awkward.

We agree the sentence as 448-449 is misplaced, this paragraph has been rewritten. We further added the sentence “We also compare performance to performing the test using only the largest site within a region.” to the first paragraph of “Efficacy of Federated Surveillance”.

Minor comments

Minor comments regarding phrasing, typos, and clarity have all been addressed, thank you for your attention to detail.
