# Supplementary material for: Federated epidemic surveillance
Source: PLoS Comput Biol. 2025 Apr 8;21(4):e1012907. doi: 10.1371/journal.pcbi.1012907 (PMC11978105; doi:10.1371/journal.pcbi.1012907)
Supplement: Supplementary file 2 [file pcbi.1012907.s005.docx]

The README.md documents things well. It would help to have a slightly more verbose description of the command-line arguments here for the scripts that use them. For example, what does “share” mean in this context?

Some notes of issues encountered while working through the code:

1. **Power Simulations**:

This seems like “dir_name” is not “tested for existence / made” anywhere, my naïve folder didn’t have it.

```
brylew@Soluna powerSimu % python calibratePthr.py --share 2 --repeats 10

Traceback (most recent call last):
  File "/Users/brylew/github/FederatedEpidemicSurveillance/powerSimu/calibratePthr.py", line 116, in <module>
    with open(os.path.join(dir_name, 's'+str(share)+meth[0]+'_'+meth[1]+'.csv'), 'a+') as df_out:
FileNotFoundError: [Errno 2] No such file or directory: 'test_bs_pthr/s2.0Stouffer_unweighted.csv'
```

I created the folder and it executes fine. Many other of the scripts provide a command-line parameter for an output folder, but this doesn’t. Given this takes a considerable period of time (more than a couple hours on my Apple Silicon laptop) to run, maybe worth echoing to stdout a message to this point, and marking progress, eg “(1/7) Calculating Stouffers” etc.

**powerCombine.py**

This doesn’t seem to run either, think it has an assumption of using a “share” value of 8 in it, the example uses 2 and thus it doesn’t find the data it wants.

```
brylew@Soluna powerSimu % python powerCombine.py --repeats 10
Traceback (most recent call last):
  File "/Users/brylew/github/FederatedEpidemicSurveillance/powerSimu/powerCombine.py", line 134, in <module>
    recArr = np.array(pd.read_csv(os.path.join(dir_name, 's'+str(share)+meth[0]+'_'+meth[1]+'.csv'), index_col=None, header=None)).mean(axis = 0)
  File "/Users/brylew/opt/anaconda3/lib/python3.9/site-packages/pandas/util/_decorators.py", line 311, in wrapper
    return func(*args, **kwargs)
  File "/Users/brylew/opt/anaconda3/lib/python3.9/site-packages/pandas/io/parsers/readers.py", line 678, in read_csv
    return _read(filepath_or_buffer, kwds)
  File "/Users/brylew/opt/anaconda3/lib/python3.9/site-packages/pandas/io/parsers/readers.py", line 575, in _read
    parser = TextFileReader(filepath_or_buffer, **kwds)
  File "/Users/brylew/opt/anaconda3/lib/python3.9/site-packages/pandas/io/parsers/readers.py", line 932, in __init__
    self._engine = self._make_engine(f, self.engine)
  File "/Users/brylew/opt/anaconda3/lib/python3.9/site-packages/pandas/io/parsers/readers.py", line 1216, in _make_engine
    self.handles = get_handle(  # type: ignore[call-overload]
  File "/Users/brylew/opt/anaconda3/lib/python3.9/site-packages/pandas/io/common.py", line 786, in get_handle
    handle = open(
FileNotFoundError: [Errno 2] No such file or directory: 'test_bs_pthr/s8.0Stouffer_unweighted.csv'
```

Like the others, it would be good to check for this before failing. It seems that there are some hard-coded “s-values” expected for this version of the code, perhaps the code should find what is available or the instructions indicate how to modify it to accept the values the user may have generated. Otherwise this “combine” function won’t produce anything.

2. **Real Data Analysis**

Like the above, while the filename hints at what the code is doing, it might be worth adding some print statements to inform user what is happening / what files have been written etc.

genSum_claim.py – several warnings appeared due to divide by 0, this is fine

claim_lag.py – Many many warnings appear related to divide by 0

genSum_hospitalization.py – many different warnings, some related to divide by 0, some related to “Mean of empty slice”

hospitalization_lag.py – Many matrices are printed to screen and many warnings appear related to divide by 0, suppressing these prints will be helpful for the user

hospitalization_otherEvidence.py – several warnings about invalid values in sqrt, “Mean of empty slice”, and “invalid value encountered in double_scalars”

I understand that these are simple warnings that happen due to data elements missing for parts of the overall data and largely are ignored / don’t affect the outcomes. If you plan to further disseminate these methods, I suggest capturing these cases and gracefully handling them in the code itself (eg if != 0 then divide, else continue (or set value to NA etc.)

3. **Semi-Synthetic Analysis**:

This relies on a conda environment it seems which I don’t have (conda activate struct)

I tried the command-line since the listed dependencies are satisfied in my native environment and got an error because the parameterized python command line in semiSyn.sh doesn’t exist.

```
brylew@Soluna semiSyn % python semiSyn.py --growthRateThr 30 --pval_thr 0.05 --nLoops 50 --maxPthr 1 --dir_name /net/dali/home/mscbio/rul98/semiSyn
python: can't open file '/Users/brylew/github/FederatedEpidemicSurveillance/semiSyn/semiSyn.py': [Errno 2] No such file or directory
brylew@Soluna semiSyn % ls
all_states.csv			semiSyn_allocShare.py		semiSyn_numSites.py
semiSyn.sh			semiSyn_amplification.py
```

This may simply be that the “shell script” semiSyn.sh didn’t get updated in the repo or that the .py was left out of the repo.

4. **Plot Scripts**

I tried to generate some plots but those scripts assume you are in the realData folder, which is not obvious till you get the error message. Once executed from within the realData folder it seems some files are not available. I am not sure if this is because I didn’t get other things to run earlier or not.

Example:

```
brylew@Soluna realData % python ../plot/plot_lag.py
Traceback (most recent call last):
  File "/Users/brylew/github/FederatedEpidemicSurveillance/realData/../plot/plot_lag.py", line 112, in <module>
    recallDf = pd.read_csv(os.path.join(dir_name, 'updating'+str(updating_period)+'training'+str(training_period)+'lag'+str(share_lag)+meth[0]+'_'+meth[1]+'_recall.csv'), index_col=None, header=None)
  File "/Users/brylew/opt/anaconda3/lib/python3.9/site-packages/pandas/util/_decorators.py", line 311, in wrapper
    return func(*args, **kwargs)
  File "/Users/brylew/opt/anaconda3/lib/python3.9/site-packages/pandas/io/parsers/readers.py", line 678, in read_csv
    return _read(filepath_or_buffer, kwds)
  File "/Users/brylew/opt/anaconda3/lib/python3.9/site-packages/pandas/io/parsers/readers.py", line 575, in _read
    parser = TextFileReader(filepath_or_buffer, **kwds)
  File "/Users/brylew/opt/anaconda3/lib/python3.9/site-packages/pandas/io/parsers/readers.py", line 932, in __init__
    self._engine = self._make_engine(f, self.engine)
  File "/Users/brylew/opt/anaconda3/lib/python3.9/site-packages/pandas/io/parsers/readers.py", line 1216, in _make_engine
    self.handles = get_handle(  # type: ignore[call-overload]
  File "/Users/brylew/opt/anaconda3/lib/python3.9/site-packages/pandas/io/common.py", line 786, in get_handle
    handle = open(
FileNotFoundError: [Errno 2] No such file or directory: 'rewrite_lag_hospitalization/updating12training12lag1wFisher_default_recall.csv'
```
